# Potentilla Canadensis

**Published:** 1877-08-20

**Authors:** 


POTENTILLA CANADENSIS.—The potentilla is a plant resembling the strawberry, growing in old fields and blooming in July or August, having a small yellow blossom. It has been called cinquefoil, or mock strawberry. The eclectics claim wonderful virtues for it as a febrifuge—particularly in typhoid fever. It is given freely in infusion.

